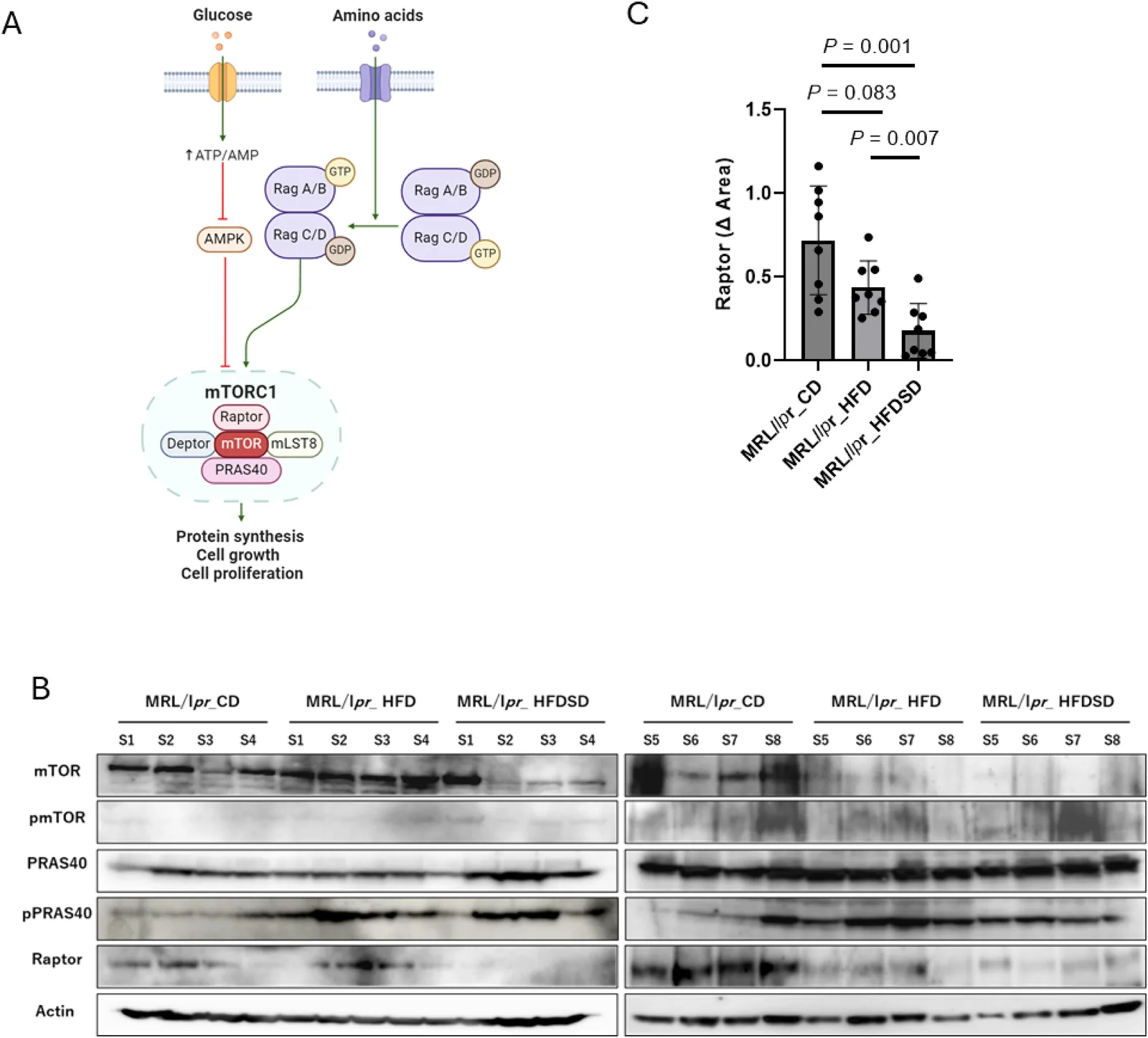# Maternal high-fat diet intake ameliorates the progression of nephritis in male lupus-prone offspring via mTOR/NFκB/arachidonic acid signaling cascade

**DOI:** 10.1016/j.gendis.2026.102216

**Published:** 2026-04-25

**Authors:** Shuang Liu, Matome Imai, Yasuyuki Suzuki, Takashi Sugiyama, Masaki Mogi

**Affiliations:** a Department of Pharmacology, Ehime University Graduate School of Medicine, Shitsugawa, Toon, Ehime 791-0295, Japan; b Department of Obstetrics & Gynecology, Ehime University School of Medicine, Shitsugawa, Toon, Ehime 791-0295, Japan; c Department of Anesthesiology, Saiseikai Matsuyama Hospital, Matsuyama, Ehime 791-8026, Japan; d Research Division, Saiseikai Research Institute of Health Care and Welfare, Minato-Ku, Tokyo 108-0073, Japan

Systemic lupus erythematosus (SLE) involving lupus nephritis (LN) predominantly affects women of childbearing age and is associated with a range of adverse pregnancy outcomes. These include not only pregnancy complications and childbirth-related issues in mothers but also health effects extending from infancy through adulthood in the next generation. Recognizing the critical impact of the intrauterine environment on metabolic balance and its close interplay with immune system development,^1^ we aimed to clarify how fetal exposure to maternal hyperlipidemia influenced disease progression in offspring predisposed to SLE. Building on our prior work demonstrating strong associations between maternal high-fat diet (HFD) exposure and alterations in SLE-relevant pathways (Fig. S1), this study tested the hypothesis that maternal dietary lipidemia critically modulates LN progression in susceptible offspring.^2^ Using a maternal HFD model in LN-prone mice, we observed reduced LN severity in offspring exposed to maternal HFD. This was supported by functional and histological analyses of kidney tissue and systemic inflammatory markers. Moreover, maternal dietary intervention appears to induce a paradigm shift in LN progression in offspring. Multi-omics analyses suggest that a potential mTOR/NFκB/arachidonic acid signaling cascade may be pivotal in modulating systemic immune responses via alterations in the fetal microenvironment. This *in vivo* study supports the hypothesis that maternal diet-driven changes in the fetal environment critically influence LN susceptibility later in life.

To clarify the effect of maternal fat intake on offspring risk of LN, we used the LN-prone MRL/lpr mouse model, which spontaneously develops severe autoimmune disease resembling human SLE, for phenotypic evaluation. All animal experimental protocols were designed and performed in accordance with the guidelines of the Animal Care Committee of Ehime University and approved by the University Committee for Animal Research (approval No. 05KI39-1). LN-prone offspring exposed to different intrauterine environments, maternal HFD (MRL/lpr_HFD), HFD during early gestation with optimized diet in late gestation (MRL/lpr_HFDSD), and normal control diet (MRL/lpr_CD), were assessed to examine the impact of maternal diet. Proteinuria onset was observed in MRL/lpr_CD offspring starting at 9 weeks of age, indicating LN initiation (Fig. 1A). Notably, both MRL/lpr_HFD and MRL/lpr_HFDSD groups exhibited significantly lower urinary protein levels than the MRL/lpr_CD group between 11 and 12 weeks. Histological analyses of 12-week-old MRL/lpr_CD mice revealed significant mesangial expansion, immune cell infiltration, and capsular adhesion in glomeruli, alongside marked C3 and IgG deposition characteristic of LN (Fig. 1B and C; Fig. S2). In contrast, MRL/lpr_HFD and MRL/lpr_HFDSD offspring displayed reduced glomerular C3 and IgG deposition.

**Figure 1:**
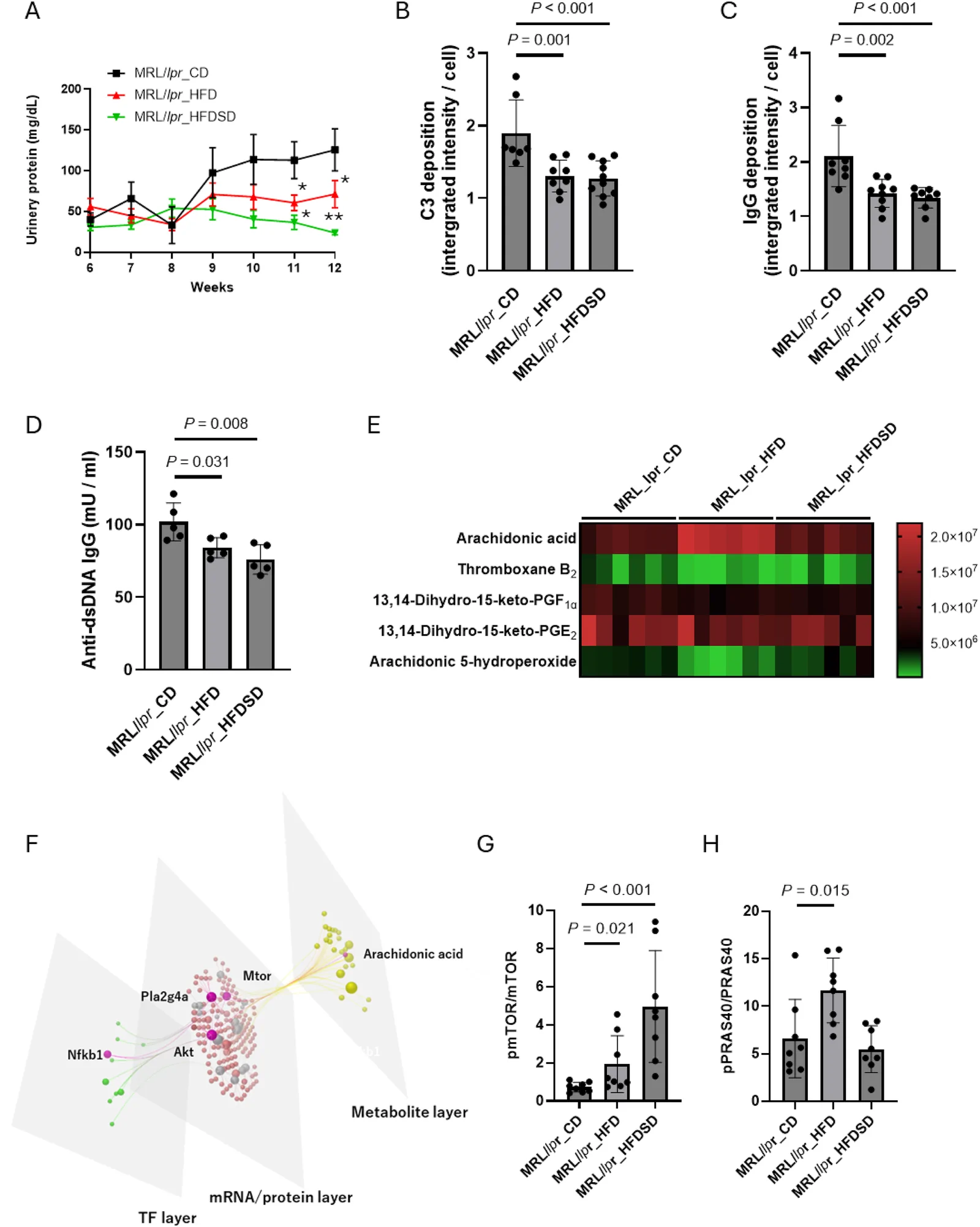
Influence of maternal fat intake on progression of lupus nephritis in lupus-prone offspring. **(A)** Proteinuria monitoring from age 6 weeks–12 weeks in MRL/lpr_CD, MRL/lpr_HFD, and MRL/lpr_HFDSD offspring (*n* = 15–32). **(B, C)** In histological analysis, quantification of (B) C3-positive area and (C) IgG-positive area in immunofluorescence staining was shown (*n* = 5–8). **(D)** Circulating levels of anti-dsDNA IgG autoantibody in the peripheral blood of 12-week-old offspring (*n* = 5). Then, the changes of metabolites in the serum of 12-week-old MRL/lpr_HFD, MRL/lpr_CD, and HFD_SD offspring were identified. **(E)** Relative quantification of arachidonic acid and its major metabolites, including prostaglandins/thromboxane and arachidonic 5-hydroperoxide, was summarized using a heatmap (*n* = 6). **(F)** Gene-metabolite interaction network was explored using the OmicsNet 2.0 platform. A multi-layer network was constructed, integrating 20 transcription factors (TFs), 48 target genes associated with the SLE signaling pathway, and 28 metabolites. Finally, key components involved in mTORC1 formation, including mTOR, phosphorated mTOR (pmTOR), PRAS40, phosphorated PRAS40 (pPRAS40), and Raptor, were assessed by Western blotting. Expression of proteins was normalized against the actin level. **(G, H)** Ratio of expression of (G) pmTOR/mTOR and (H) pPRSA40/PRSA40 was quantified (Δarea: normalized area against actin level; *n* = 8). Results were presented as mean ± standard deviation.

We further quantified circulating anti-dsDNA IgG autoantibodies in peripheral blood at 12 weeks (Fig. 1D). Both MRL/lpr_HFD and MRL/lpr_HFDSD offspring showed significantly lower serum anti-dsDNA IgG levels compared with MRL/lpr_CD controls. Additionally, cytokine profiling revealed that maternal HFD exposure was associated with mitigated LN risk in SLE-prone offspring (Fig. S3).

To comprehensively evaluate the potential effects of maternal HFD exposure on disease onset in SLE-prone offspring, we performed bulk RNA sequencing on spleen samples from offspring exposed to different intrauterine environments (Fig. S4). Differentially expressed genes within the SLE signaling pathway were extracted. Notably, *Mtor* expression was significantly increased in MRL/lpr_HFD offspring, whereas *Nfkb1* and *Pla2g4a* expression were suppressed relative to MRL/lpr_CD offspring. Liquid chromatography-mass spectrometry-based untargeted metabolomics was also conducted to identify serum metabolite changes in offspring (Fig. S5). Among the top 15 metabolites contributing most to LN prediction accuracy, arachidonic acid (MDA = 0.0031) emerged as a key biomarker potentially predictive of outcomes linked to maternal high-fat exposure. Relative quantification of arachidonic acid and its major metabolites, including prostaglandins, thromboxanes, and arachidonic 5-hydroperoxide, was achieved by integrating chromatographic peak data. Heatmap analysis revealed elevated serum arachidonic acid levels and distinct alterations in its metabolite profile in MRL/lpr_HFD offspring compared with MRL/lpr_CD and MRL/lpr_HFDSD groups (Fig. 1E).

To investigate underlying mechanisms by which maternal lipidemic exposure influences SLE progression, we constructed a comprehensive gene–metabolite interaction network using OmicsNet 2.0. A multi-layer network integrating 20 transcription factors, 48 target genes in the SLE pathway, and 28 metabolites revealed robust interconnectivity, with *Nfkb1* emerging as a central regulatory hub linking the mTOR signaling pathway to arachidonic acid metabolism (Fig. 1F). Specifically, *Nfkb1* was associated with the up-regulation of key genes such as *mTOR* and *Akt1*, critical for SLE onset and progression, while also being linked to the down-regulation of *Pla2g4a* encoding phospholipase A2. This was quantitatively supported by a log2(fold change) of −1.514 between MRL/lpr_HFD and MRL/lpr_CD. The observed multi-omics patterns are consistent with an mTOR → Akt1 → Nfkb1 → Pla2g4a → arachidonic acid-associated axis, linking inflammatory signaling with arachidonic acid metabolism. However, the directionality and regulatory hierarchy cannot be inferred from these correlative data alone. As an initial exploration, we assessed key mTORC1 components at the protein level^3^ (Fig. S6). Phosphorylated mTOR levels were significantly increased in MRL/lpr_HFD mice and were further elevated in MRL/lpr_HFDSD mice relative to MRL/lpr_CD (Fig. 1G). A distinct pattern was observed for phosphorylated PRAS40 (pPRAS40), a direct inhibitor of mTORC1 substrate binding (Fig. 1H). An elevated pPRAS40/PRAS40 ratio was detected in MRL/lpr_HFD offspring but not in MRL/lpr_HFDSD offspring compared with the MRL/lpr_CD group. These findings reveal an mTOR/Nfkb1/arachidonic acid cascade that appears to modulate disease progression in SLE-prone offspring exposed to a maternal lipidemic environment, highlighting a complex regulatory mechanism warranting further study.

The influence of an HFD-shaped intrauterine environment on the paradigm shift observed in LN-prone offspring remains incompletely understood. By integrating transcriptomic and metabolomic analyses, we identified a potential molecular signature involving the mTOR/NFκB/arachidonic acid cascade, among other pathways, associated with disease activity. Notably, arachidonic acid has recently been identified as a circulating metabolomic biomarker negatively correlated with SLE disease activity.^4^ In line with clinical observations, our study revealed higher serum arachidonic acid levels and lower levels of its downstream metabolites, alongside the down-regulation of *Pla2g4a* transcript levels, in LN-improved, HFD-exposed offspring compared with controls. These findings suggest functional modulation of phospholipase activity induced by maternal HFD exposure. As a sensor of energy sufficiency and environmental stress, fetal mTOR activity may directly or indirectly regulate the expression of protein phosphatase 2A through known Akt/mTORC1-NFκB crosstalk, potentially contributing to the observed alterations in phospholipase function.^5^ Confirming this mechanistic link will require further investigation of mTORC1, NFκB, protein phosphatase 2A, and phospholipase family member activities to elucidate the molecular signatures underlying HFD-induced changes in disease progression.

In conclusion, our *in vivo* functional study provides evidence supporting the data-driven hypothesis that the fetal microenvironment plays a critical role in predisposing individuals to LN later in life. We identified a novel mTOR/NF-κB/arachidonic acid axis that appears to mediate key inflammatory and metabolic processes in LN pathogenesis. Our findings underscore the potential long-term benefits of early nutritional and environmental interventions. By exploring genetic and epigenetic modifications during early development, it may be possible to reduce disease susceptibility not only in affected individuals but also in their offspring. Such intergenerational benefits could help break the cycle of inherited predisposition to autoimmune diseases, offering a transformative approach to early prevention and improved health outcomes for future generations.

## CRediT authorship contribution statement

**Shuang Liu:** Writing – original draft, Project administration, Methodology, Data curation, Conceptualization. **Matome Imai:** Writing – review & editing, Methodology, Data curation. **Yasuyuki Suzuki:** Writing – review & editing, Software, Data curation. **Takashi Sugiyama:** Writing – review & editing, Supervision, Funding acquisition. **Masaki Mogi:** Writing – review & editing, Validation, Supervision, Funding acquisition.

## Ethics declaration

All animal experimental protocols were designed and performed in accordance with the guidelines of the Animal Care Committee of Ehime University and were approved by the University Committee for Animal Research (approval No. 05KI39-1).

## Funding

This work was funded by the Grant-In-Aid for Scientific Research from the Ministry of Education, Science, and Culture of Japan (No. 22K08564 to M.M., 24K11579 to S.L., 21K16549 to Y.S.) and the Smoking Research Foundation of Japan. The funders had no role in study design, data collection and analysis, decision to publish, or preparation of the manuscript.

## Conflict of interests

The authors have no competing interests to declare.

## Figures and Tables

**figs1:**
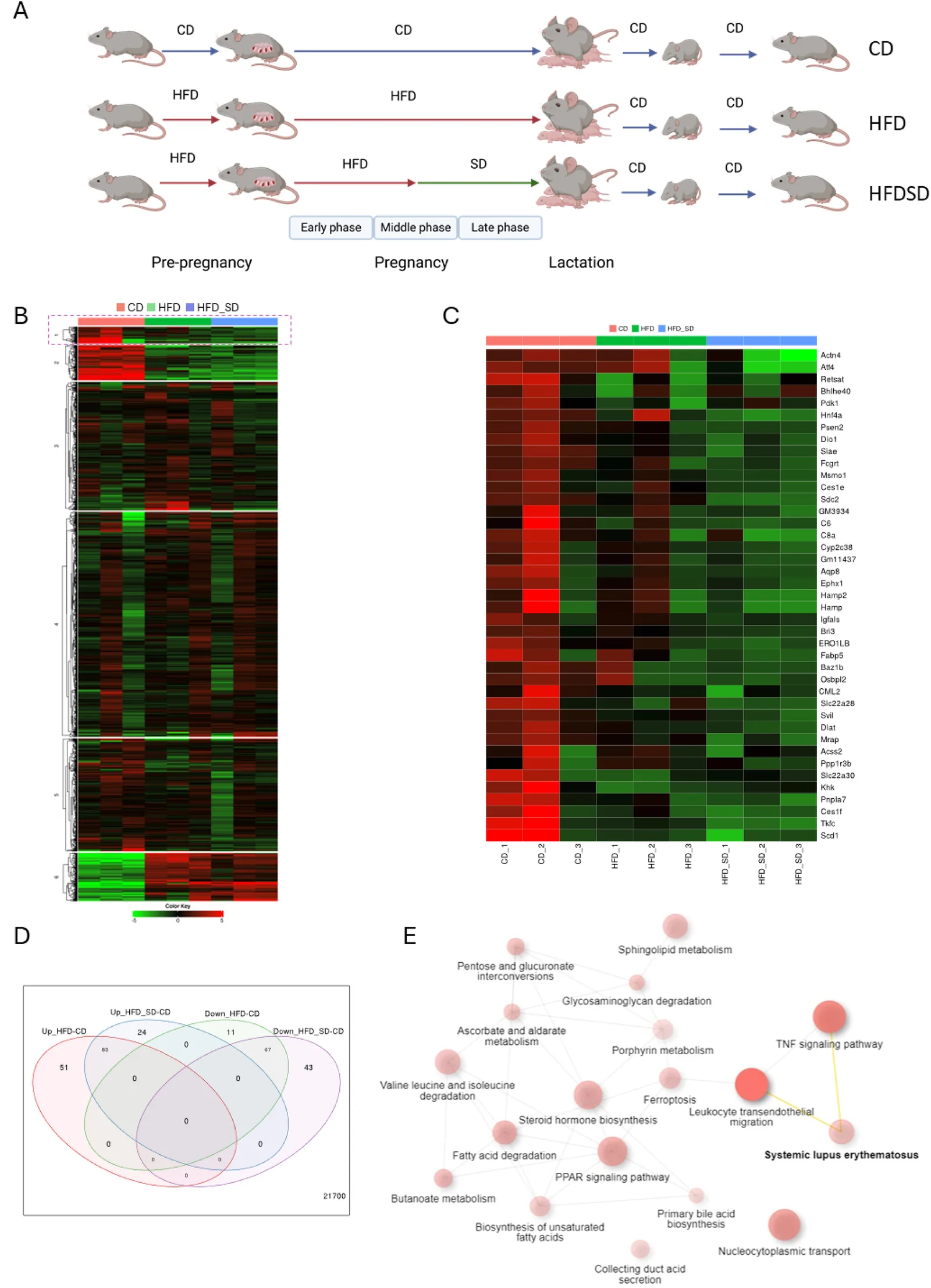


**figs2:**
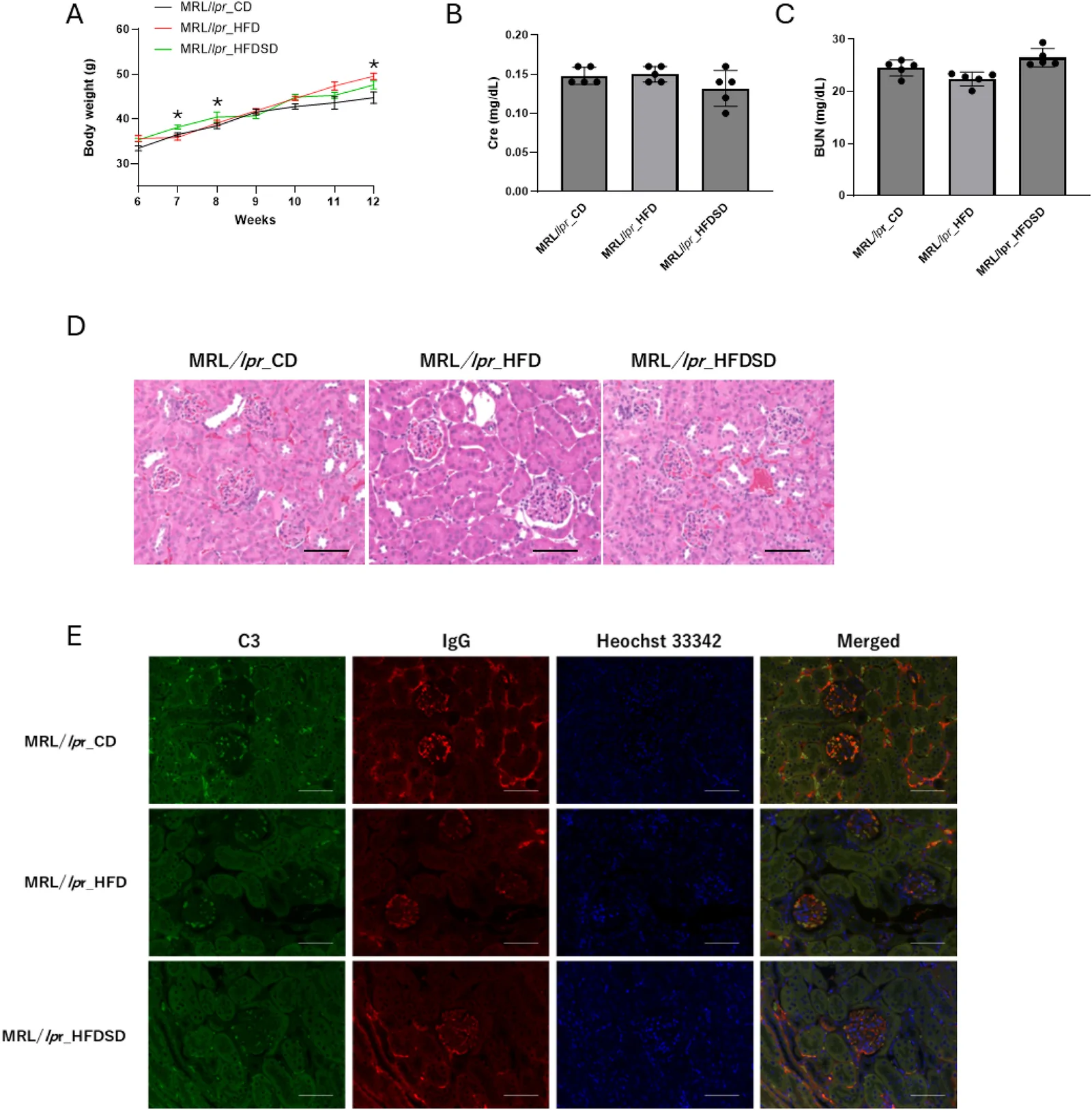


**figs3:**
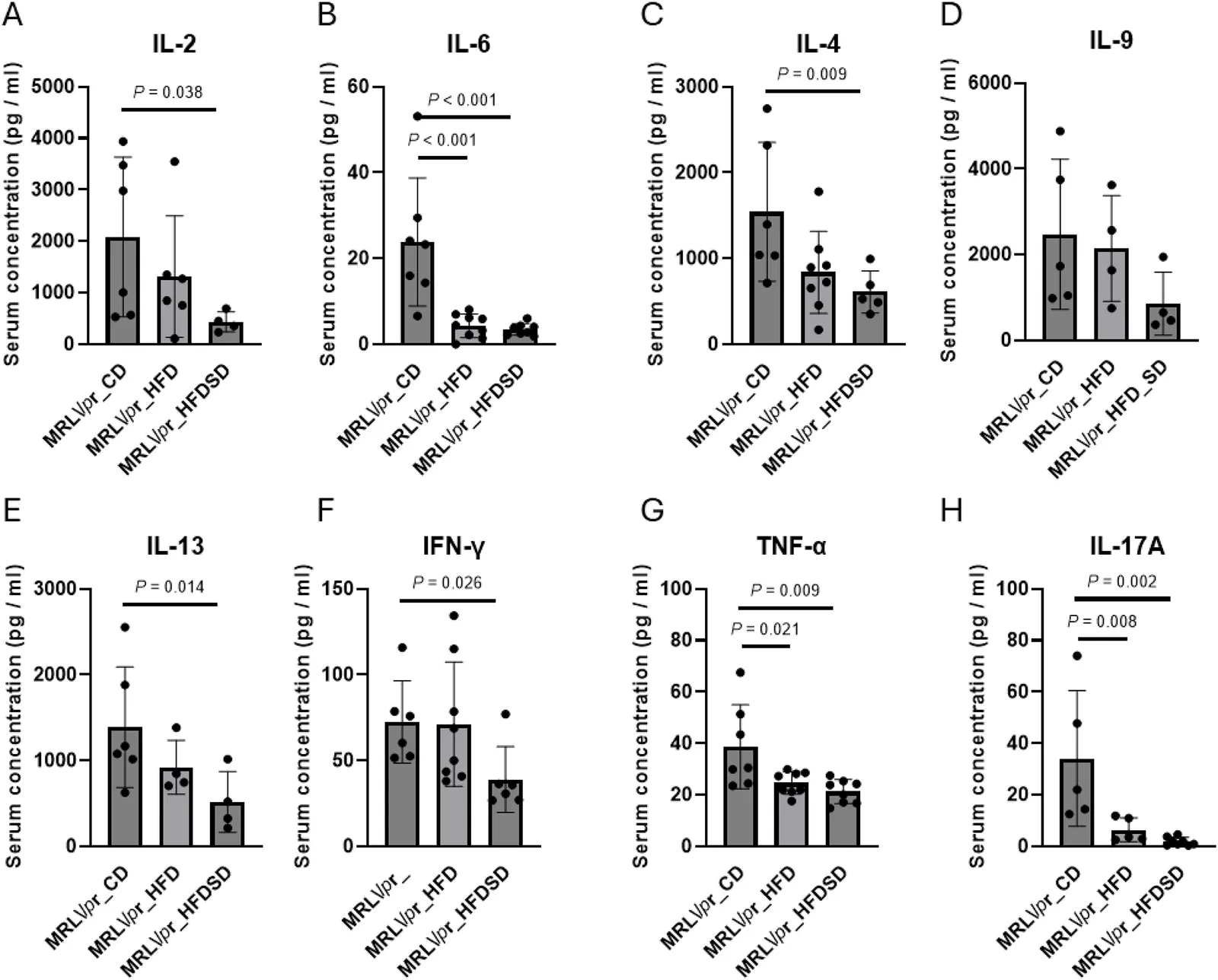


**figs4:**
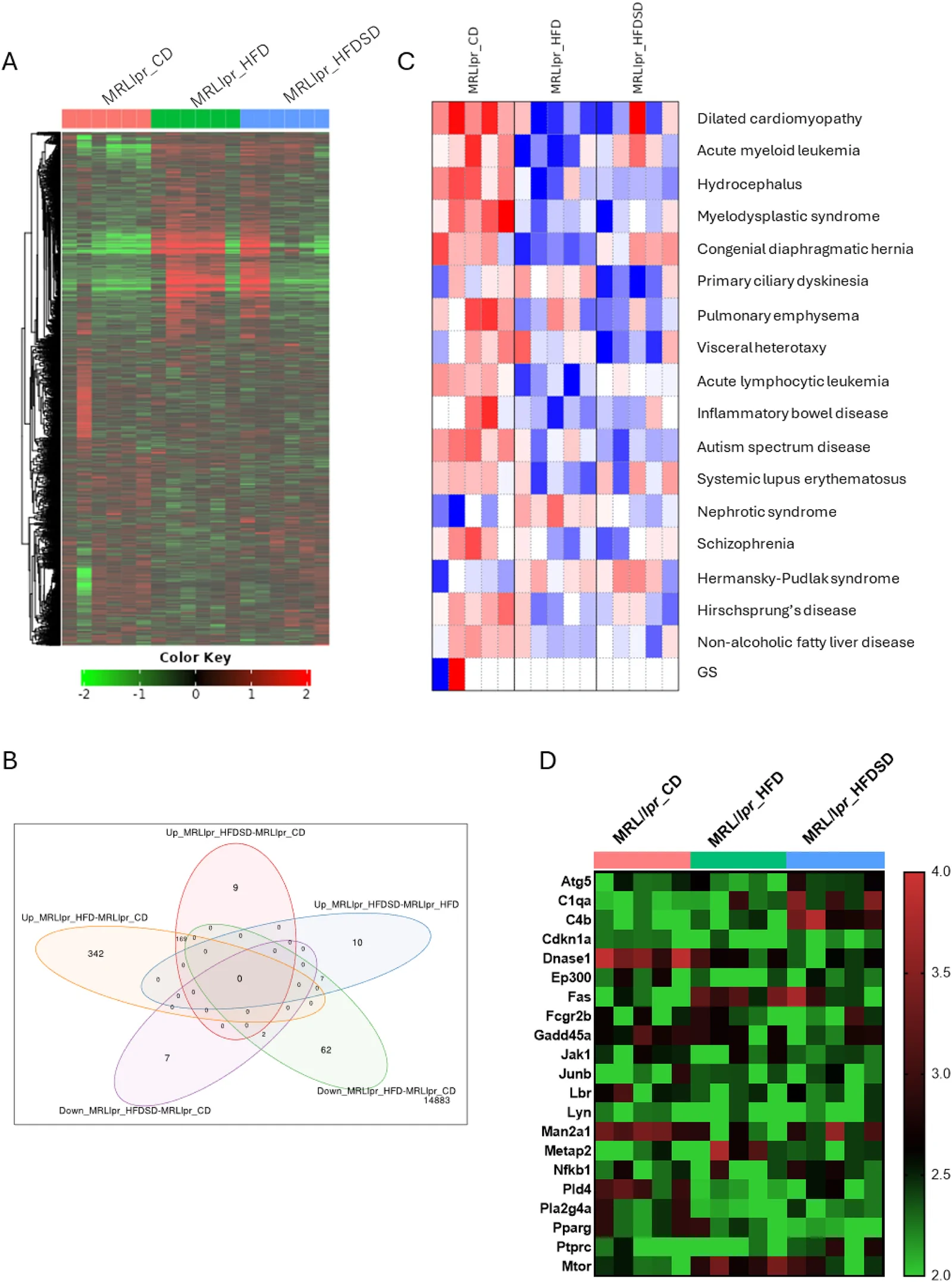


**figs5:**
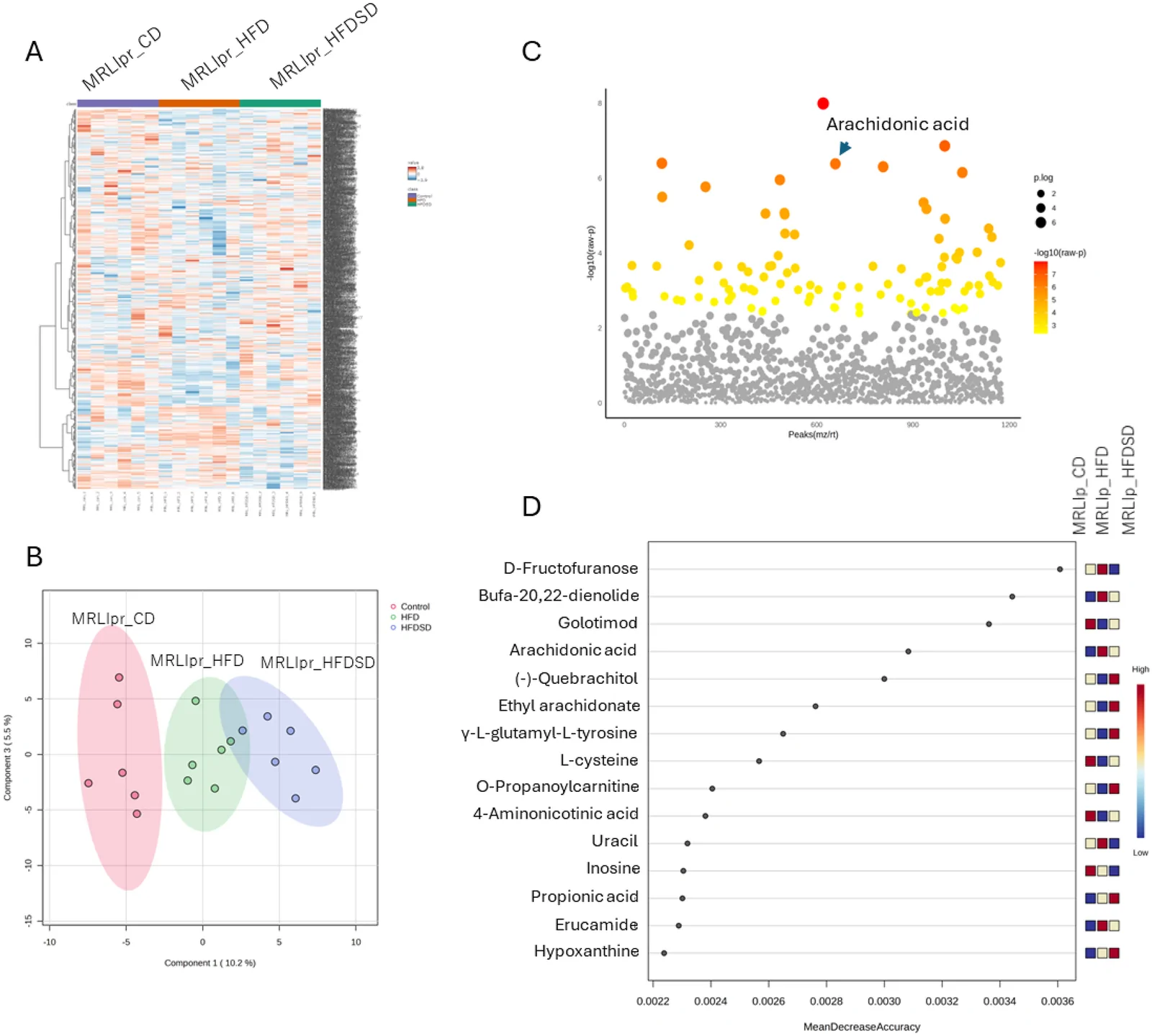


**figs6:**