# Supplementary material for: Maternal high-fat diet intake ameliorates the progression of nephritis in male lupus-prone offspring via mTOR/NFκB/arachidonic acid signaling cascade
Source: Genes Dis. 2026 Apr 25;14(2):102216. doi: 10.1016/j.gendis.2026.102216 (PMC13628648; doi:10.1016/j.gendis.2026.102216)
Supplement: Supplementary file 1 [file mmc1.docx]

**Methods and materials**

**Animals**

All animal experimental protocols were designed and performed in accordance with the guidelines of the Animal Care Committee of Ehime University and approved by the University Committee for Animal Research (approval No. 05KI39-1).

Five-week-old C57BL6/N (Clea Japan, Tokyo, Japan) or MRL/MpJJmsSlc-*lpr/lpr* (Japan S.LC., Shizuoka, Japan) (MRL/lpr) mice, a model to determine the etiology of SLE, were maintained in a temperature-controlled room (22 ± 2°C) under specific pathogen-free conditions, with a 12-h light-dark cycle.

The maternal high-fat intake model was established according to our previous studies with sight modification. Female mice were assigned to receive either a control diet (CD; 3.85 kcal/g), comprising 14% fat, 57% carbohydrate, and 29% protein (formula D12450B, Research Diets, New Brunswick, NJ, USA), or a high-fat diet (HFD; 4.73 kcal/g), consisting of 45% fat, 35% carbohydrate, and 20% protein (formula D12451, Research Diets), for a period of 4 weeks. Age-matched male mice were maintained on CD starting at 5 weeks of age and utilized for mating at 9 weeks of age. Detection of a vaginal plug in female mice was designated as gestational day 0 (G0). Pregnant females continued on their respective diets (HFD or CD) until gestational day 10 (G10). Following G10, the pregnant mice were segregated into three experimental groups: (1) a control group that remained on CD, (2) an HFD group that continued on HFD, and (3) a nutritional intervention group that transitioned from HFD to CD after G10. Postpartum, all groups were uniformly maintained on CD.

After birth, the body weights and the progression of proteinuria were monitored every 7 days from 6-12 weeks of age. Offspring were subjected to peripheral blood chemistry screening at the age of 12 weeks.

**Transcriptomics**

Offspring (n = 3-6 mice / group) were euthanized and tissues were dissected out for RNA extraction. Total RNA was isolated using a NucleoSpin RNA Plus kit (TaKaRa, Tokyo, Japan). After RNA quality assessment on a Bioanalyzer 2100 system (Agilent Technologies, Tokyo, Japan), samples with RIN value of 7.0 or higher were selected for subsequent transcriptome analysis.

For microarray analysis, newborns (n= 3 mice/group) and total RNA were extracted from the liver lobes of newborns. RNA was fragmented using the Gene Chip Sample Cleanup Module (Affymetrix, Sunnyvale, CA, USA) and labeled with a single color using the Agilent enlarge notation method. Hybridization, staining, washing, and scanning procedures were carried out as described in the Gene Chip Expression Analysis technical manual (Affymetrix).

For RNA-seq, total RNA was extracted from the spleen of male offspring (12 weeks old). Nanopore sequencing was carried out using a ligation-based cDNA-PCR sequencing kit (Oxford Nanopore Technologies, Didcot, UK) according to the manufacturer’s protocol. Briefly, using the strand-switching protocol, full-length transcripts were amplified and the adaptor-ligated libraries were sequenced using a MinION sequencer with FLO-MIN 106 flow cells and R9.4 chemistry. Basecalling was performed using a Guppy basecaller (version 6.4.6+ae70e8f) by translating ionic signals into a nucleotide sequence. Subsequently, Minimap2 software (version 2.24-r1122) was employed to align the FASTQ files to SAM files with the reference genome generated from GCF_000001635.27_GRCm39_genomic.gtf (External item). The SAM files were then converted to BAM files, sorted, and indexed using Samtools software (version 1.15.1). Finally, FeatureCounts (version 2.0.4) was utilized to perform read counting using the Mus_musculus.GRCm39.109.gtf annotation file.

**Metabolomics**

Peripheral blood of male offspring (12 weeks old) was collected from the submandibular vein by quick puncture with a lancet. Metabolomic analysis was undertaken as described previously. Briefly, metabolites were extracted by adding 95 μl 80% methanol into a 5 μl serum sample. After 2 h incubation on ice and centrifugation, the supernatant was collected and dried using an evacuated centrifuge. After dissolving in MilliQ water, the samples were applied to an Orbitrap Exploris 240 Mass Spectrometer coupled with a Vanquish UHPLC system (Thermo Fisher Scientific, Tokyo, Japan). Separation was performed on a Hypersil GOLD™ aQ C18 column (Thremo Fisher Scientific).

The mobile phase consisted of solvent A (0.1% formic acid in MilliQ water) and B (0.1% formic acid in methanol), applied at a flow rate of 0.3 mL/min. The gradient program was carried out with 0-50% B at t=0-8 min; 50-98% B at t=8-9 min; 98% B at t=9-14 min; and 0% B at t=14-15 min. The column temperature was maintained at 45℃.

Mass spectrometry analysis was performed in electrospray ionization (ESI) mode, operating in both positive and negative ionization modes. Spray voltage was 3.5 kV for positive mode and 2.5 kV for negative mode. Capillary temperature was set at 275°C, and vaporizer temperature was maintained at 350°C. Sheath gas and auxiliary gas flow rates were set to 50 and 10 arbitrary units. Data acquisition was conducted over a mass range of m/z 70–800, with resolution of 120,000 at m/z 200. Full scan MS data were collected with data-dependent MS/MS using a normalized collision energy of 15, 30, and 45%. Data processing was carried out using a Compound Discoverer 3.3 (Thermo Fisher Scientific).

**Integrated bioinformatics**

Bioinformatic analysis was conducted by integrating RNA-seq and metabolomics data to gain comprehensive insights into the biological mechanisms under study. Raw read counts or normalized expression data of transcriptomes were obtained, and identification of differentially expressed genes (DEGs) was performed using a cloud platform for integrated differential expression and pathway analysis (iDEP1.1; http://bioinformatics.sdstate.edu/, accessed on 20 April, 2023) by extraction using DESeq2 with an FDR cutoff of 0.1 and min-fold change of 2 as the default setting.

Metabolomics data were processed using MetaboAnalyst, a comprehensive tool for data normalization, statistical analysis, and functional interpretation. Raw metabolomics data were uploaded to MetaboAnalyst 6.0 (<https://www.metaboanalyst.ca/>, accessed on 4 September, 2024), where data cleaning, auto-scaling, and normalization were conducted. Multivariate statistical analyses, including Principal Component Analysis (PCA) and Partial Least Squares Discriminant Analysis (PLS-DA), were used to identify patterns and potential biomarkers as the default setting.

Integrated pathway analysis was carried out using OmicsNet 2.0 to visualize the connections between transcriptomics and metabolomics data. DEGs included in the SLE signaling pathway and significant metabolites were uploaded into OmicsNet 2.0, which utilized KEGG and Reactome databases to map and analyze metabolic and signaling pathways. Network analysis was performed to identify interactions and enriched pathways, highlighting potential mechanistic links between transcriptomic factors, gene expression and metabolic changes.

Cytokine profiling and anti-double strained DNA (dsDNA) antibody measurement in serum

Serum of male offspring (12 weeks old) was collected for cytokine and anti-dsDNA IgG assessment (n = 5-8). Cytokine levels in serum were quantified by a bead-based immunoassay using a LEGENDplex™ Mouse Th Cytokine Panel (BioLegend Japan KK, Tokyo, Japan), including IL-2, -6, -4, -9, -13, -17A, IFN-γ, and TNF-α, according to the manufacturer’s instructions. Labelled beads were measured using a BD FACSCalibur system (BD Biosciences, San Jose, CA, USA), and acquired data were analysed using web-based LEGENDplex data analysis software (Biolegend).

Serum levels of dsDNA-antibody were quantified using a LBIS^TM^ mouse dsDNA-IgG ELISA kit (Fujifilm, Tokyo, Japan), according to the manufacturer’s instructions. Briefly, serum was incubated in a dsDNA-coated plate to capture anti-dsDNA antibody. Horseradish peroxidase-labeled anti-mouse IgG antibody was used as a secondary antibody, and chromogen colorimetric development was performed. Absorbance of the product is measured spectrophotometrically at 450nm. Anti-dsDNA IgG concentration was determined using standard curve methods (assay range: 15.6 – 1000 mU / ml).

**Histological analysis**

Kidneys were harvested from mice, fixed in 4% paraformaldehyde, and embedded in paraffin. To assess SLE-associated glomerulonephritis and mononuclear cell infiltration, sections were stained with hematoxylin and eosin. For semiquantitative analysis of IgG and C3 deposition in glomeruli, sections were stained with anti-mouse IgG conjugated to Alexa Fluor 488 (ThermoFisher Scientific) and anti-mouse C3 IgG (Abcam, Cambridge, UK). Hoechst 33342 (Dojindo, Kumamoto, Japan) was used for nuclear counterstaining. Positive staining areas, representing IgG or C3 accumulation, were segmented from background objects. For each specimen, five high-power fields per section and three sections were analysed, and the ratio of integrated fluorescence intensity to area per cell was calculated for normalized quantification (n = 6–8). Imaging and analysis were conducted using an all-in-one microscope (BZ-X800; Keyence, Tokyo, Japan).

**Western blotting**

To measure protein expression levels in the mTOR signaling pathway, western blotting was performed following a standard protocol. Briefly, fresh spleens were collected from 12-week-old male offspring, then homogenized in RIPA buffer (Thermo Fisher Scientific) supplemented with HALT protease and phosphatase inhibitors (Thermo Fisher Scientific). Spleen lysates were separated on 5-20% gradient gels (ATTO, Osaka, Japan) and transferred to a polyvinylidene difluoride membrane using the iBlot 2 dry blotting system (Thermo Fisher Scientific). Membranes were incubated with specific primary antibodies, followed by horseradish peroxidase-conjugated secondary antibodies. Primary antibodies included anti-mTOR (7C10), phospho-mTOR (D9C2), Raptor (S792), PRAS40 (D23C7), and phospho-PRAS40 (T246), all from Cell Signaling Technology (Danvers, MA). Expression of β-actin in lysates was detected using a monoclonal anti-mouse-β-actin antibody (AC-74) (Merck, Tokyo, Japan) for normalization of expression levels. Blots were developed using a chemiluminescent substrate and imaged with an Amersham ImageQuant800 system (Cytiva, Tokyo, Japan).

**Statistical analysis**

All statistical analyses were performed using GraphPad Prism 10 (GraphPad Software, La, Jolla, CA). All experiments were designed in a completely randomized multifactorial format. Sample distributions were analyzed using the Kolmogorov-Smirnov test. Comparisons between two groups were performed using unpaired non-parametric Mann-Whitney test. Comparisons among multiple groups were performed by two-way ANOVA, followed by Tukey's multiple comparison test, for the data of body weight monitoring and urinary protein level. Data were presented as mean ± standard error. Differences with P < 0.05 were considered statistically significant.
