# Supplementary material for: Maternal high-fat diet intake ameliorates the progression of nephritis in male lupus-prone offspring via mTOR/NFκB/arachidonic acid signaling cascade
Source: Genes Dis. 2026 Apr 25;14(2):102216. doi: 10.1016/j.gendis.2026.102216 (PMC13628648; doi:10.1016/j.gendis.2026.102216)
Supplement: Supplementary file 2 [file mmc2.docx]

**Supplementary Figure legends**

S. Fig. 1 Association between maternal fat-intake and SLE signaling pathway in offspring of wild mice. (A) Workflow of diet-intake in investigation. (B) Heatmap and κ-means clustering revealed six major clusters of the top 2000 expressed genes in CD, HFD, and HFDSD offspring. Expression is displayed as log_2_ (fold-change) (n= 3). Enrichment analysis (KEGG) revealed that Cluster 1 (red dotted line) was highly correlated with the SLE signaling pathway. (C) Comparison of expression levels of DEGs involving the SLE signaling pathway shared among CD, HFD, HFDSD offspring. Values are log_2_-transformed expression of DEGs. (D) Venn diagram showing number of upregulated and downregulated genes in comparisons of HFD vs. CD and HFD_SD vs. CD, identified by transcriptomic analysis. (E) Network of enriched pathway by gene ontology enrichment analysis in the comparison of HFD vs CD offspring (cut off = 0.3).

S. Fig. 2 Influence of maternal fat-intake on progression of LN in lupus-prone offspring. (A)Body weight monitoring from age 6 weeks to 12 weeks in MRL/lpr_CD, MRL/lpr_HFD and MRL/lpr_HFDSD offspring (n = 15-32). Serum levels of (B) BUN and (C) creatine were measured in 12-week-old offspring (n = 5). Typical histological images of kidneys including (D) hematoxylin and eosin staining and (E) immunofluorescence staining of C3 and IgG glomerular deposition in MRL/lpr_CD, MRL/lpr_HFD and MRL/lpr_HFDSD offspring (scale bar = 50μm) (n= 5). Results are presented as mean ±SD.

S. Fig. 3 Cytokine profiles associated with autoantibody production and systemic inflammation in serum, including (A) IL-2, (B) IL-6, (C) IL-4, (D) IL-9, (E) IL-13, (F) IFN-γ, (G) TNF-α, and (H) IL-17A, determined in 12-week-old MRL/lpr_CD, MRL/lpr_HFD and MRL/lpr_HFDSD offspring using a LEGENDplex Cytokine panel (n= 5-8). Results are presented as mean ±SD.

S. Fig. 4 Transcriptome analysis in splenocytes of LN-prone offspring. (A) Heatmap analysis revealed the top 2000 expressed genes in MRL/lpr_CD, MRL/lpr_HFD, and MRL-/lpr_ HFDSD offspring. Expression is displayed as log_2_ (fold-change) (n = 5). (B) Venn diagram showing number of upregulated and downregulated genes (DEGs) identified by transcriptomic analysis in comparisons among MRL/lpr_HFD, MRL/lpr_CD and HFD_SD offspring. (C)Pathway analysis (PGSEA) using the Disease.MGI database predicted disease onset risks in SLE-prone offspring. Enrichment analysis (KEGG) revealed that Cluster 1 (red dotted line) was highly correlated with the SLE signaling pathway. (D) Comparison of expression levels of DEGs involving in SLE signaling pathway shared among MRL/lpr_HFD, MRL/lpr_CD and HFD_SD offspring. Values are log_2_-transformed expression of DEGs.

S. Fig. 5 Identify changes in metabolites in serum of LN-prone offspring. (A) Heatmap of levels of 1,914 identified metabolites in serum of 12-week-old MRL/lpr_HFD, MRL/lpr_CD and HFD_SD offspring (n = 6). (B) The identified metabolites were subjected to partial least squares discriminant analysis. Divergent serum metabolomic profiles among groups were revealed. (C) ANOVA followed by post hoc analysis identified 144 significantly altered metabolites among the MRL/lpr_CD, MRL/lpr_HFD, and MRL/lpr_HFDSD groups. (D) The predictive importance of these metabolites in distinguishing outcomes between groups was assessed using mean decrease accuracy from Random Forest analysis. The top 15 metabolites contributing most significantly to prediction accuracy were ranked.

S. Fig. 6 Crosstalk of mTOR/Nfkb/arachidonic acid axis in maternal high-fat-exposed offspring. (A) Schema of mTORC1-related signaling pathway which is potentially influenced by energy sufficiency input. (B) Key components involved in mTORC1 formation, including mTOR, phosphorated mTOR (pmTOR), PRAS40, phosphorate PRAS40 (pPRAS40), and Raptor were assessed by western blotting. Expression of proteins was normalized against actin level. (C) Ratio of expression of Raptor quantified (∆area: normalized area against actin level; n = 8). Results are presented as mean ±SD.
